# Computational Characterizations of the Interactions Between the Pontacyl Violet 6R and Exoribonuclease as a Potential Drug Target Against SARS-CoV-2

**DOI:** 10.3389/fchem.2020.627340

**Published:** 2021-01-21

**Authors:** Rangika Munaweera, Ying S. Hu

**Affiliations:** Department of Chemistry, College of Liberal Arts and Sciences, University of Illinois at Chicago, Chicago, IL, United States

**Keywords:** exoribonuclease, SARS-CoV-2, pontacyl violet 6R, DEDDh exonucleases, RNA-dependent RNA polymerase, orthocoronavirinae

## Abstract

We report a molecular-docking and virtual-screening-based identification and characterization of interactions of lead molecules with exoribonuclease (ExoN) enzyme in severe acute respiratory syndrome coronavirus 2 (SARS-CoV-2). From previously identified DEDDh/DEEDh subfamily nuclease inhibitors, our results revealed strong binding of pontacyl violet 6R (PV6R) at the catalytic active site of ExoN. The binding was found to be stabilized *via* two hydrogen bonds and hydrophobic interactions. Molecular dynamics simulations further confirmed the stability of PV6R at the active site showing a shift in ligand to reach a more stabilized binding. Using PV6R as the lead molecule, we employed virtual screening to identify potential molecular candidates that form strong interactions at the ExoN active site. Our study paves ways for evaluating the ExoN as a novel drug target for antiviral treatment against SARS-CoV-2.

## Introduction

Due to the highly contagious nature of the virus and the high cost associated with the production of synthetic RNA molecules, the traditional high throughput screening has not shown sufficient effectivity in identifying new drugs against the severe acute respiratory syndrome coronavirus 2 (SARS-CoV-2). To this end, computational studies play critical roles by narrowing down potential hits through accurate theoretical predictions, such as using molecular docking and molecular dynamics simulations. This study employed computational techniques to identify lead molecules that competitively bind at the exoribonuclease (ExoN) site of non-structural protein 14 (nsp14) from SARS-CoV-2.

The RNA-dependent RNA polymerase (RdRp) is a critical enzyme that enables the error-prone replication of RNA viruses and facilitates the rapid adaptation of these viruses to changing circumstances (Crotty et al., 2001; Elena and Sanjuan, 2005). RNA synthesis by the RdRp is one of the main drug targets in the formulation of antiviral agents for genetically diverse *Orthocoronavirinae* viruses (CoVs) (Brown et al., 2019). *In vitro* studies have demonstrated the efficacy of nucleoside analogs (NuA) in CoV infected cells (Warren et al., 2016; Jordan et al., 2018; Pruijssers and Denison, 2019; Tchesnokov et al., 2019). Specifically, intracellular kinases metabolize NuA to the corresponding 5′-triphosphates of the NuA (Nu3P), thus perturbing endogenous nucleoside triphosphate pools (Warren et al., 2016). RdRp incorporates Nu3Ps to the growing RNA strands, resulting in mutated RNA products. Accumulation of mutated RNA products leads to lethal mutagenesis for the viruses, thereby achieving the desired antiviral effect (Vignuzzi et al., 2005).

**FIGURE 1:**
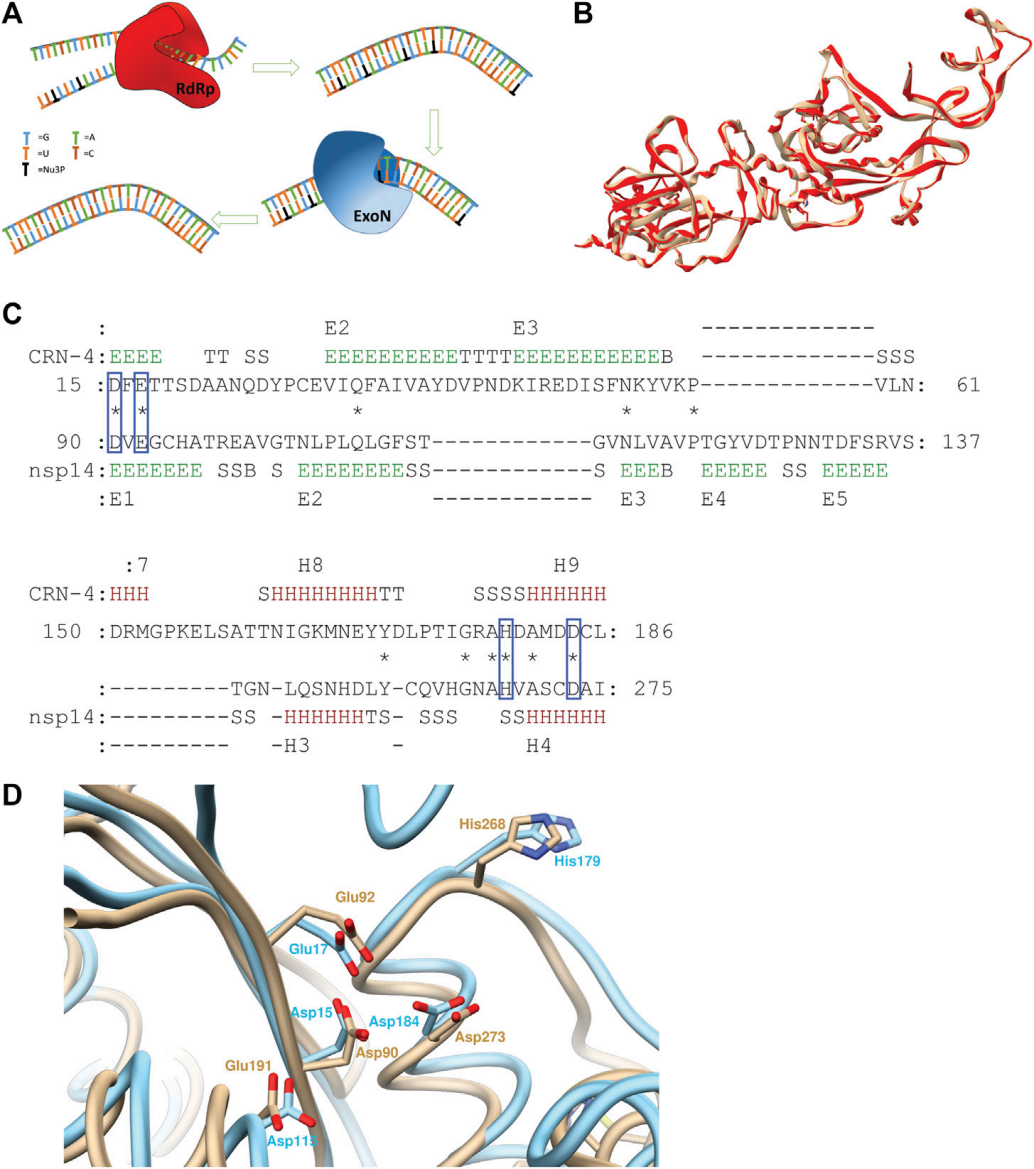
**(A)** Schematic illustration of ExoN activity countering the drug effect of nucleoside analogs. **(B)** Superimposed structures of 5NFY template (beige) and homology model (red) **(C)** Pairwise 3D structure alignment of active sites of CRN-4 and homology model of nsp14 indicating the alignment of homologues catalytic residues of two enzymes in blue boxes (regions indicated by letter E show extended strands which participates in beta ladders; regions indicated by letter T show hydrogen bonded turns; regions indicated by letter S show bends; regions indicated by letter B show residues in isolated beta-bridges; regions indicated by letter H show alpha helices) **(D)** CRN-4 (cyan) and nsp14 (beige) superimposed highlighting active site residues. 90th aspartic acid, 92nd glutamic acid, 191th glutamic acid, 268th histidine, 273rd aspartic acid residues of nsp14 aligns with 15th aspartic acid, 17th glutamic acid, 115th aspartic acid, 179th histidine and 184 aspartic residues of CRN-4 respectively. SARS-CoV-2 has a 191th glutamic acid residue while 115th CRN-4 has an aspartic acid residue.

However, the effectiveness of NuAs, such as the nucleotide prodrug remdesivir (GS-5734), against SARS-CoV-2 can be countered by the presence of exoribonuclease (ExoN) enzymes. In particular, the ExoN enzymes proofread erroneously added Nu3Ps in the RNA products (Figure 1A) (Minskaia et al., 2006; Eckerle et al., 2010; Smith et al., 2013; Agostini et al., 2018). This mechanism counters the effect of NuAs and may lead to the requirement of a higher dosage of NuAs with increased systemic side-effects (Agostini et al., 2018; Dong et al., 2020).

CoVs contain one of the largest viral genomes ranging from 27 to 34 kB. The viral genome encodes for both structural (sp) and nonstructural proteins (nsp). Out of the 16 nsps, nsp12 is known as the RdRp. The RdRp forms a complex with two other nsps (nsp7 and nsp8) to function in the cellular environment (Kirchdoerfer and Ward, 2019; Ogando et al., 2019). Nsp 14, also known as the ExoN, is classified into the superfamily of DEDD exonucleases. DEDD exonucleases contain the proofreading domains of many DNA polymerases as well as other eukaryotic and prokaryotic exonucleases (Ogando et al., 2019). Further, structural studies of SARS-CoV nsp14 showed the presence of five catalytic residues. These features reveal that nsp14 is a member of DEDDh/DEEDh subfamily (Huang et al., 2016; Ogando et al., 2019). Interestingly, nsp14 in SARS-CoV exhibits both the (N7-guanine)-methyltransferase (N7-MTase) activity and ExoN activity. N7-MTase domain is located in the C-terminal domain of nsp14. Studies confirmed that these two enzymatic activities (ExoN and N7-MTase) of the nsp14 are functionally distinct and physically independent, although they are structurally interconnected (Ogando et al., 2019).

Importantly, the proofreading activities of ExoN may counter the drug effect of Nu3Ps (Ogando et al., 2019). A recent report argued that an effective anti-viral drug design has to consider the balancing act between nsp12 and nsp14 (Shannon et al., 2020). Despite being a potential drug target, few compounds have been identified to inhibit the nsp14 activity *in vitro* (Huang et al., 2016). Simultaneous attack of RdRp and nsp14 using a combination of a nucleoside analog and a specific nsp14 inhibitor may enhance lethal mutagenesis in SARS-CoV-2 (Ogando et al., 2019). This strategy may enable the use of a range of NuAs that have been identified as possible drugs but failed to show efficacy *in vitro* and *in vivo*.

Since the nsp14 structure of SARS CoV-2 has not been solved at the time of this study, a homology model was constructed using the nsp14 of SARS CoV as the template. Candidate inhibitor molecules were computationally docked to the modeled nsp14 protein to identify potential lead molecules. Identified molecules were further evaluated for their stability using molecular dynamics (MD) simulations. Interactions of the strongly binding ligand molecule were computationally characterized and its molecular features were extracted. Virtual screening was performed using the extracted molecular features from lead molecule. Further, we optimized lead molecules using a number of computational strategies.

## Materials and Methods

### Homology Modeling and Active Site Alignment

The newly-emerged SARS-CoV-2 nucleotide gene (YP_009725309.1) was retrieved from the National Center for Biotechnology Information (NCBI) nucleotide database (https://www.ncbi.nlm.nih.gov/protein/YP_009725309.1). A homology model for the SARS-CoV-2 nsp14 was built using the Swiss Model web server (Waterhouse et al., 2018). The structure of the SARS-CoV nsp10/nsp14 complex (PDB ID: 5NFY) was the most sequelogous (99% sequence identity) to the SARS-CoV-2 nsp14 with a resolution of 3.38 Å. The quality of the homology model was analyzed using QMEAN score and Ramachandran plot generated by Swiss Model server itself (Waterhouse et al., 2018). It was further analyzed using the ProSA server (Wiederstein and Sippl, 2007). The active site pairwise structure alignment of CRN4 and homology model was carried out using TM align server and MATRAS protein 3D structure comparison tool (Kawabata, 2003; Zhang and Skolnick, 2005).

### Molecular Docking

Molecular docking studies were performed using the AutoDock Vina software package. Results were analyzed using Autodock Tools and Chimera packages (Forli et al., 2016; Anil, 2019). Blind dock studies were carried out using previously identified three DEDDh/DEEDh subfamily nuclease inhibitors, pontacyl Violet 6R (PV6R), aurintricarboxylic acid (ATA) and 5,5′-dithiobis (2-nitrobenzoic acid) (DTNB) (Huang et al., 2016). The homology model of the SARS-CoV-2 nsp14 was used as the macromolecule.

### Molecular Dynamics Simulations

MD simulations were performed using GROMACS software package. The RMSD of the PV6R molecule at the ExoN active site was calculated. Docking pose of the PV6R molecule was used to carry out the MD simulation. Ligands topology was prepared using CGenFF server and protein topology was prepared using Gromacs software itself. MD simulations were done using CHARMM36 all-atom force field. All other molecule preparations were done using Chimera software package unless otherwise stated.

Prepared complexes were immersed in a cubic box with a 1.5 nm distance between the protein surface and the boundary of the cubic box. The selected cubic box was filled with SPC/E water molecules to solvate the system and periodic boundary conditions were applied from all sides. To neutralize the protein and to maintain the net ionic strength of the system to a value closer to the physiological conditions Na^+^ and Cl^−^ ions were added replacing the solvent molecules. Energy minimization was done using the steepest descent algorithm with an energy minimization step of 0.01 kJ mol^−1^ for 50,000 steps setting the maximum force value for 1,000 kJ mol^−1^ nm^−1^. NVT and NPT equilibrations were carried out for a total of 100 ps for each with 2 fs steps. Product MD run was performed for a total of 21.0 ns consist of 2 fs steps. Temperature and pressure coupling were performed using the modified Berendsen thermostat and Parrinello-Rahman method. Ewald particle mesh method was used to calculate long-range electrostatic interactions. Results were analyzed using the VMD software package.

### Virtual Screening, Lead-like Molecule Identification, Physiochemical and Pharmacokinetics Studies

The compound selected from molecular docking (lead compound) was virtually screened using the OpenEye Scientific vROCS application(ROCS 3.3.2.2: OpenEye Scientific Software, Santa Fe, NM. http://www.eyesopen.com.; Hawkins et al., 2007) Virtual screening was carried out using eMolecule and Kishida databases (ROCS 3.3.2.2: OpenEye Scientific Software, Santa Fe, NM. http://www.eyesopen.com). Best 20 molecules from each database were docked with homology model and average structure obtained from MD simulation using Autodock vina and the binding energies of selected compounds were calculated. Compounds that bind at the ExoN active site were selected. Control docks were carried out with endogenous ribonucleotides. All of the compounds were prepared to be optimized in their active forms in physiological conditions using Avagadro software (López, 2012). Binding of selected compounds at the ExoN active site of nsp14 was further examined using LigPlot application (Wallace et al., 1995). ADME parameters, pharmacokinetic properties, druglike nature and medicinal chemistry friendliness was evaluated using SwissADME server (Daina et al., 2017).

The lead molecule was used as the seed compound to generate novel compounds through generative shape-based neural network decoding using the LigDream web application (Skalic et al., 2019). The PV6R molecule was used as the seed compound to generate novel compounds from generative shape-based neural network decoding. Novel compounds generated by LigDream were arranged using RDock software based on the RDock score (Ruiz-Carmona et al., 2014). Five compounds with the best RDock score were directed to molecular docking using AutoDock vina for comparison purposes.

## Results and Discussions

### Homology Modeling and Active Site Alignment

The SWISS MODEL server was used to build the homology model for the SARS-CoV-2 nsp14 using SARS-CoV nsp14/nsp10 complex (PDB ID: 5NFY) as the template. The template had a 99% sequence alignment with SARS-CoV-2 nsp14. The built homology model showed a QMEAN score of −3.18, GMQE score of 0.98, and MolProbity results were better compared to the template (Supplementary Table S1). ProSA server results showed a Z-score of -9.15, suggesting an accurate homology model. The Produced model was further evaluated using the TM align server and MATRAS pairwise 3D alignment web server shown in Supplementary Data 1 (Kawabata, 2003; Zhang and Skolnick, 2005). A 98% structure alignment (512 residues align with template out of 523 residues in the entire protein) of the homology model was obtained within a five Å range compared to the template and a root mean square deviation (RMSD) of 0.15 Å (Figure 1B). These results suggest a high-quality homology model, based on which our studies have been performed. The Ramchandran plots of the Homology model and the template are provided in Supplementary Figures S1, S2.

PV6R, ATA and DTNB have been previously identified as CRN-4 of and RNase T inhibitors. CRN-4 and RNase T are DEDDh exonucleases found in *C. elegans* and *E. coli,* respectively (Huang et al., 2016). Structure alignment of CRN-4 and nsp14 of SARS-CoV-2 using MATRAS pairwise 3D structure alignment and Chimera structure comparison shows close proximity (Figures 1C,D) of active site residues. Specifically, all DEDDh exonucleases share a conserved active site with a common set of residues (Huang et al., 2016; Ogando et al., 2019). In the nsp14 of SARS-CoV-2, residues Asp 90, Glu 92 (motif I), Glu 191 (motif II), Asp 273 (motif III) and His 268 (motif III) constitute the active site while the equivalent of E191 alternates between E and D in different nidovirus taxa. Nsp14 has Asp 90, Glu 92, Glu 191, His 268 and Asp 273 residues as the active site residues while CRN-4 having Asp15, Glu 17, Asp 115, His 179 and Asp 184 as active site residues (Huang et al., 2016; Ogando et al., 2019). This observation suggests that previously identified inhibitors for the DEDDh exonuclease may serve as effective inhibitors of nsp14.

### Molecular Docking

**FIGURE 2:**
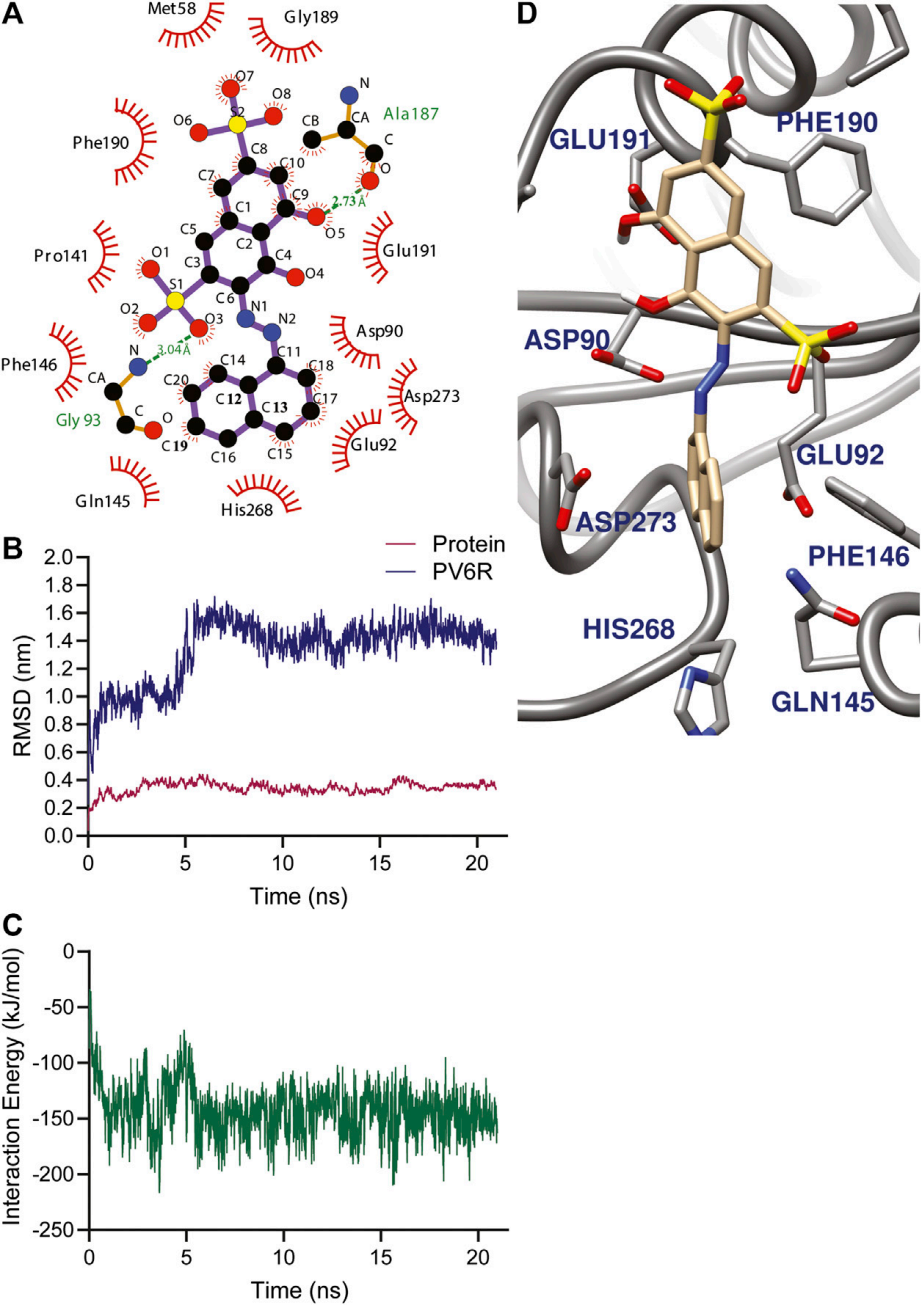
**(A)** Amino acid environment of the PV6R molecule bound to the active site of nsp 14. PV6R forms two hydrogens bonds with 187th Alanine residue and 93rd Glycine residue. It is further stabilized at the binding site through hydrophobic interactions including all the active site residues; Asp 90, Glu 92, Glu 191, His 268 and Asp 273. MD simulations results showing **(B)** RMSD of protein backbone and PV6R at ExoN site during the simulation time (21.0 ns) showing a shift in binding type achieving better interaction energy according to **(C)** Interaction energy (kJ/mol) plot of PV6R with active site residues during 21.0 ns simulation **(D)** Snapshot of simulation taken at 10 ns.

Using the selected DEDDh exonucleases inhibitors, molecular docking revealed that PV6R binds to the ExoN binding site of nsp14 with a preferable calculated binding energy of -8.3 kcal/mol. We further analyzed the interactions between active site residues and PV6R. Figure 2A shows that the PV6R molecule is stabilized *via* two hydrogen bonds at the active site including a very short hydrogen bond between Ala 187 and fifth oxygen of PV6R along with hydrophobic interactions significantly contributing to the stabilization. Interestingly, most of these hydrophobic interactions are formed with five residues (Asp 90, Glu 92, Glu 191, His 268 and Asp 273) that are involved in the catalytic activity. Protonated state of PV6R shows a similar calculated binding energy (−8.3 kcal/mol) forming hydrogen bonds with Gly 93, His 268, Asp 273 and Asn 266N.

### Molecular Dynamics Simulations

The stability of the PV6R and ExoN complex in an aqueous solution was evaluated using the parameter RMSD. MD simulation was performed for 21.0 ns using the docked structure of PV6R to ExoN. The average RMSD of the trajectories for bound protein backbone atoms showed relative stability (Figure 2B). During the simulation PV6R molecules shows a shift in binding mode with structural conformational change to achieve more stabilized interaction at the ExoN active site showing more stable calculated interaction energy in the later part of the simulation (Figures 2B–D). This flexibity is observed due to the presence of more polar groups in PV6R that can form stronger interaction with acidic residue rich active site of ExoN.

### Virtual Screening and Lead-like Molecule Identification

**FIGURE 3:**
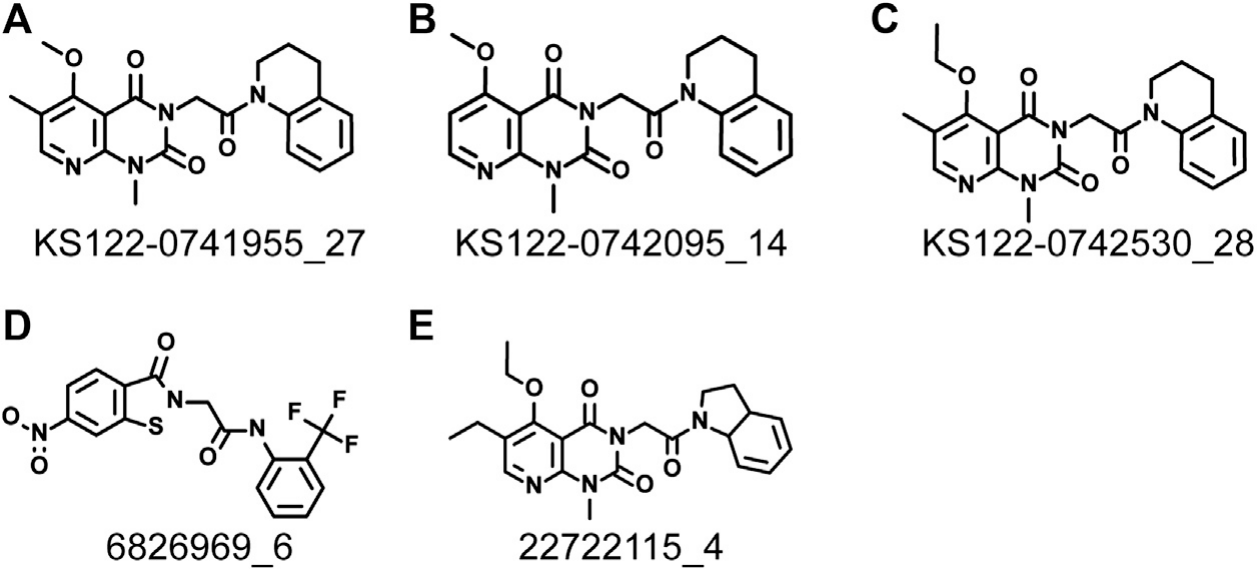
Identified molecules that show favorable binding at nsp14 active site from virtual screening **(A)** KS122-0741955_27 **(B)** KS122-0742095_14 **(C)** KS122-0742530_28 **(D)** 6826969_6 **(E)** 22722115_4.

Next, virtual screening was performed using the vROCS application (OpenEye scientific software). This software package identifies the molecular features of a given molecule based on the structure, shape and electrostatics. To identify structurally and electrostatically similar molecules, the PV6R molecule was compared with the eMolecules database and Kishida database from the OpenEye software. Parameters were set to provide 20 best fits for each run. From obtained hits, molecules that bind at the active site with a calculated binding energy of less than -7.0 kcal/mol were selected and shown in Table 1. Two molecules from the eMolecules database (Molecule reference numbers: 22722115_4 and 6826969_6) and three molecules from the Kishida database (Molecule reference numbers: KS122-0741955_27, KS122-0742530_28, KS122-0742095_14) were selected for further studies as shown in Figure 3.

**TABLE 1:** Calculated binding energies of selected ligands at ExoN active site.

	Ligand	TanimotoCombo score	Calculated binding energy with homology model (kcal/mol)	Calculated binding energy with average structure obtained from MD simulation (kcal/mol)
Lead molecules	DTNB	n/a	>−7.0	n/a
	ATA	n/a	>−7.0	n/a
	PV6R	n/a	−8.3	n/a
Virtual screening - OpenEye scientific eMolecules database	22722115_4	0.970	−7.0	>−7.0
	6826969_6	0.957	−7.6	−7.4
Virtual screening - OpenEye scientific kishida database	KS122-0741955_27	1.031	−8.2	−8.3
	KS122-0742530_28	0.999	−8.7	−9.1
	KS122-0742095_14	0.994	−7.9	−8.0
Positive control runs	ATP	n/a	−6.7	n/a
	GTP	n/a	−7.2	n/a
	CTP	n/a	−6.9	n/a
	UTP	n/a	−7.0	n/a
5 selected compounds based on Rdoc score from generative modeling (Please refer Supplementary Data for structures)	18	n/a	−7.7	n/a
	40	n/a	−7.9	n/a
	48	n/a	−7.8	n/a
	61	n/a	−7.8	n/a
	79	n/a	−7.9	n/a

**FIGURE 4:**
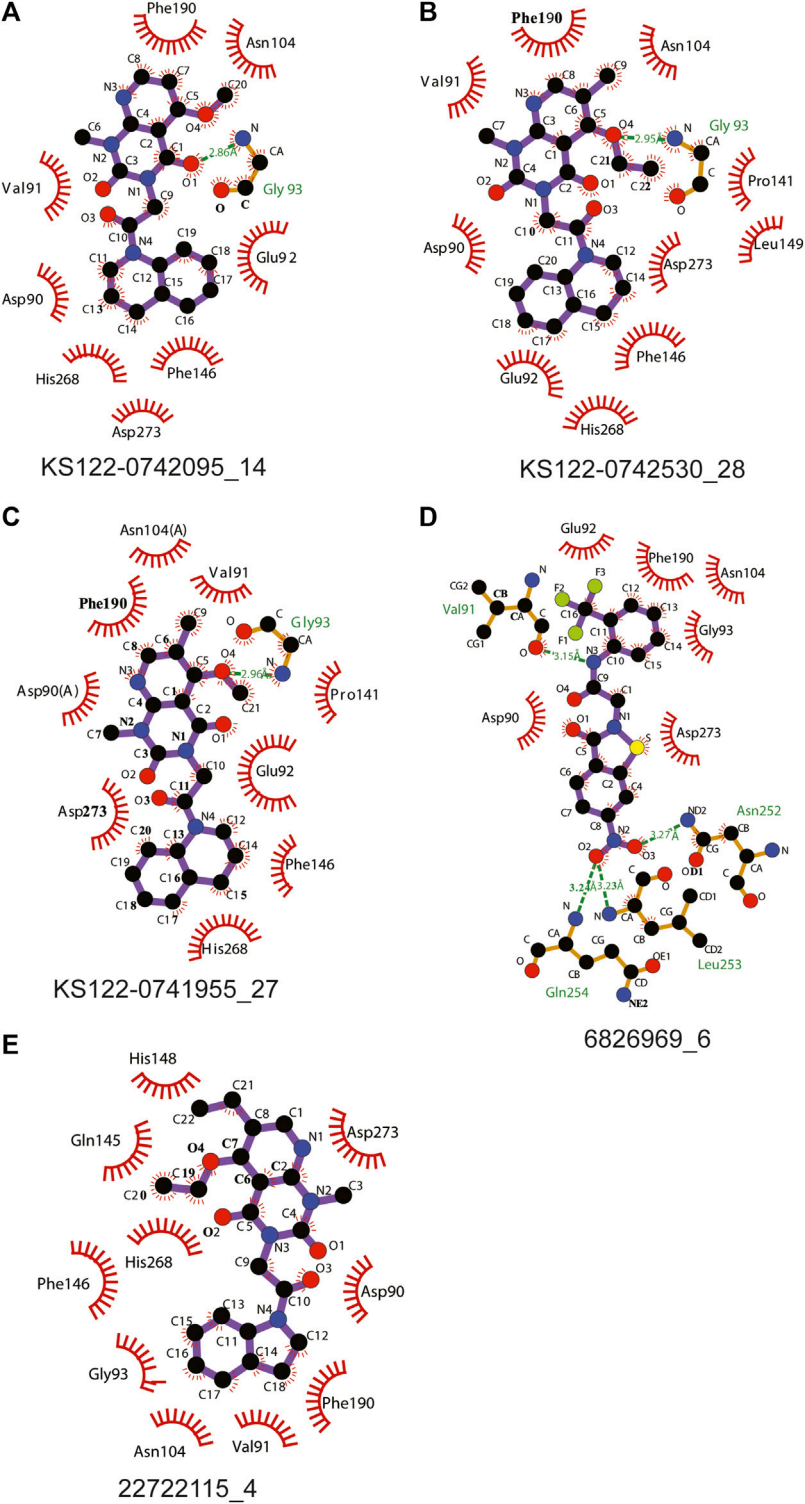
Interactions of molecules identified from virtual screening **(A)** KS122-0742095_14 **(B)** KS122-0742530_28 **(C)** KS122-0741955_27 **(D)** 6826969_6 **(E)** 22722115_4 at nsp14 ExoN active site with amino acid environment.

Figure 4 shows the molecular docking studies of these five selected compounds identified in Figure 3 (22722115_4, 6826969_6 from eMolecules database and KS122-0741955_27, KS122-0742530_28, KS122-0742095_14 from Kishida database). Each compound was further analyzed by the LigPlot application to identify the interactions that make these molecules stable at the binding site. Table 2 shows the interacting amino acids with the PV6R and the five selected compounds at the ExoN binding site. KS122-0741955_27, KS122-0742095_14 and KS122-0742530_28 exhibit a similar pattern of interactions at the active site forming one hydrogen bond with the Gly 93 residue and several hydrophobic interactions. All three molecules form hydrophobic interactions with Asp 90, Val 91, Glu 92, Pro 141, Phe 146, Phe 190, Val 191, His 268 and Asp 273 while KS122-0741955_27 and KS122-0742530_28 displays interactions with Asn 104 and KS122-0742530_28 form an extra hydrophobic interaction with Leu 149. Calculated Binding free energies of KS122-0741955_27, KS122-0742095_14 and KS122-0742530_28 were −8.2 kcal/mol, −7.9 kcal/mol and −8.7 kcal/mol, respectively.

**TABLE 2:** Interacting amino acids in the docking poses of the ligands.

Compound	Calculated binding energy (kcal/mol)	Interacting amino acids in the active site	H Bonds
PV6R	−8.3	Met 58, Asp 90, Glu 92, Gly 93, Pro 141, Gln 145, Phe 146, Ala 187, Gly 189, Phe 190, Glu191, His 268 and Asp 273	2
PV6R (Protonated)	−8.3	Met 58, Glu 92, Gly 93, Asn 104, Pro 141, Phe 146, Leu 149, Phe 190, His 268, Asp 273, Asn 266	4
KS122-0741955_27	−8.2	Asp 90, Val 91, Glu 92, Gly 93, Asn 104, Pro 141, Phe 146, Phe 190, Val 191, His 268 and Asp 273	1
KS122-0742530_28	−8.7	Asp 90, Val 91, Glu 92, Gly 93, Asn 104, Pro 141, Phe 146, Leu 149, Phe 190, His 268 and Asp 273	1
KS122-0742095_14	−7.9	Asp 90, Val 91, Glu 92, Gly 93, Pro 141, Phe 146, Phe 190, Val 191, His 268 and Asp 273	1
22722115_4	−7.0	Asp 90, Val 91, Glu 92, Gly 93, Asn 104, Phe 190, Phe 191, Asn 252, Leu 253, Gln 254, Asp 273	4
6826969_6	−7.6	Asp 90, Val 91, Gly 93, Asn 104, Gln 145, Phe 146, His 148, Phe 190, His 268, Asp 273	0

From the calculated binding energies, KS122-0742530_28 represents the most favorable candidate due to the presence of extra hydrophobic interactions along with close H bond acceptor and donor localizations (2.95 nm). Calculated binding free energy of -8.7 kcal/mol suggests KS122-0742530_28 as a better ExoN inhibitor than PV6R. Molecules identified from eMolecules database showed higher binding free energies due to the presence of high electronegative groups and the formation of weak hydrogen bonds in 6826969_6 and the lack of strong intermolecular hydrogen bonding in 22722115_4.

Molecular docking studies between compounds identified with virtual screening and average structure of ExoN obtained from MD simulation showed further decrease for the KS122-0741955_27, KS122-0742530_28 and KS122-0742095_14 molecules with a calculated binding energy value of −8.3 kcal/mol, −9.1 kcal/mol and 8.0 kcal/mol respectively. Molecules from eMolecules database showed an increase in energy, as shown in Table 1. The drastic stability enhancement of KS122-0742530_28 can be due to its structural similarity to the PV6R molecule showing similar trend as did the PV6R molecule during the MD simulation. In general, the enhanced binding strength of molecules obtained from Kishida database shows their effectiveness and can be promising lead molecules for further experimental validation.

Next, a technique based on machine learning was used to generate novel molecules using PV6R as the seed compound. Three-dimensional shape and pharmacophoric features of the seed molecule (lead molecule) were used to produce lead-like compounds (Skalic et al., 2019). 92 identified novel compounds were further arranged using the RDock binding scores at the ExoN active site of the nsp14 (SMILES of 92 compounds have been provided in Supplementary Table S2). Five compounds (Supplementary Figure S3) with the best RDock scores were further docked using AutoDock vina. Their calculated binding energies at the ExoN site were tabulated in Table 1. Together with the virtual screening results, these identified molecules with preferable binding at the Exon active site serve as promising candidates for further investigations of their competitive inhibition of nsp14.

### Physiochemical and Pharmacokinetics Studies

Physiochemical studies of the selected compounds (Table 3) showed that the calculated TPSA values were within acceptable limits. All selected compounds possess a large number of hydrogen bond acceptors and 6826969_6 has two hydrogen bond donors. The presence of moieties that enable the hydrogen bond formation increases the binding affinity at the active site of the ExoN. A higher polar surface area indicates the drug’s ability to permeate the cell membrane. Lipophilicity was calculated as an average value of iLOGP, XLOGP3, WLOGP, MLOGP and SILICOS-IT (Daina et al., 2017) indicated as log P, which can be implicated in blood-brain barrier penetration and permeability prediction. Further, log P along with the molecular weight (MW) can be used to predict the metabolism and excretion of xenobiotic compounds from the human body. All compounds possess better aqueous solubility compared to PV6R according to this analysis (Table 3).

**TABLE 3:** Physiochemical and general properties of ligands.

Compound	Oral bioavailability: TPSA (Å^2^)	MW (g/mol)	Log P	H Bonds		Solubility (ESOL)		
				No. of H bond acceptors	No. of H bond donors	Log S	Solubility (mg/ml)	Class
PV6R	196.34	472.45	2.76	10	2	−5.19	3.04 × 10–3	Moderately soluble
KS122-0741955_27	88.43	394.42	2.06	5	0	−3.42	9.45 × 10–2	Soluble
KS122-0742530_28	86.43	408.45	2.38	5	0	−3.85	5.71 × 10–2	Soluble
KS122-0742095_14	86.43	380.4	1.73	5	0	−3.31	1.84 × 10–1	Soluble
22722115_4	86.43	410.47	2.14	5	0	−3.61	1.01 × 10–1	Soluble
6826969_6	129	398.34	1.33	7	2	−3.97	4.28 × 10–2	Soluble

High gastrointestinal (GI) absorption and impermeability of blood-brain barrier (BBB) make these promising candidates to be used as drugs against respiratory illness (Table 4). The pharmacokinetic parameters suggest that these compounds follow drug-likeness rules (Lipinski, Ghose, Veber, Egan and Muegge) confirming that these molecules are drug-like and have a strong possibility to be directed for further studies to be used as drugs (Ghose et al., 1999; Egan et al., 2000; Muegge et al., 2001; Veber et al., 2002; Lipinski et al., 2012). Two medicinal chemistry filters, pan assay interference compounds (PAINS) and Brenk, showed 0 alerts for four of the selected compounds, suggesting their lead likeness.

**TABLE 4:** ADME, pharmacokinetics, drug-likeness and medicinal friendliness of ligands.

Compound	GI absorption	BBB permeant	P-gp substrate	Log Kp (skin permeation) cm/s	Druglikeness					Bioavailability score	PAINS	Brenk	Synthetic accessibility
					Lipinski	Ghose	Veber	Egan	Muegge				
PV6R	Low	No	No	−6.68	Yes	No	No	No	No	No	1	2	3.62
KS122-0741955_27	High	No	No	−7.36	Yes	Yes	Yes	Yes	Yes	0.55	0	0	3.11
KS122-0742530_28	High	No	No	−7.19	Yes	Yes	Yes	Yes	Yes	0.55	0	0	3.24
KS122-0742095_14	High	No	No	−7.54	Yes	Yes	Yes	Yes	Yes	0.55	0	0	2.97
22722115_4	High	No	No	−7.26	Yes	Yes	Yes	Yes	Yes	0.55	0	0	4.48
6826969_6	Low	No	Yes	−6.88	Yes	Yes	Yes	Yes	Yes	0.55	0	1	3.16

In summary, our molecular docking and molecular dynamics studies show that PV6R binds at the ExoN catalytic active site of SARS-CoV-2 with a strong calculated free binding energy of -8.3 kcal/mol. Virtual screening using PV6R as the lead molecule identified KS122-0742530_28 from the Kishida database with a better binding free energy. Both PV6R and KS122-0742530_28 confirmed to have drug-like physiochemical and pharmacokinetic properties. These molecules may serve as lead molecules in further experimental validation for the development of ExoN inhibitors against SARS-CoV-2, including profiling their 50% inhibitory concentration (IC50) values and virus-inhibitory effects.

## Data Availability Statement

The original contributions presented in the study are included in the article/Supplementary Material, further inquiries can be directed to the corresponding author.

## Author Contributions

All authors listed have made a substantial, direct, and intellectual contribution to the work and approved it for publication.

## Conflict of Interest

The authors declare that the research was conducted in the absence of any commercial or financial relationships that could be construed as a potential conflict of interest.
