# Supplementary material for: Computational Characterizations of the Interactions Between the Pontacyl Violet 6R and Exoribonuclease as a Potential Drug Target Against SARS-CoV-2
Source: Front Chem. 2021 Jan 21;8:627340. doi: 10.3389/fchem.2020.627340 (PMC7858249; doi:10.3389/fchem.2020.627340)
Supplement: Supplementary file 1 [file datasheet1.docx]

Supplementary Material

**Supplementary Table 1. MolProbity results comparison between template and homology model.**

|  |  | Template | Homology Model |
| --- | --- | --- | --- |
| Peptide Omegas | Poor rotamers | 2.46% | 1.31% |
|  | Favored rotamers | 96.51% | 97.39% |
|  | Ramachandran outliers | 2.69% | 1.34% |
|  | Ramachandran favored | 89.34% | 89.44% |
|  | Rama distribution Z-score | -2.30 ± 0.15 | -0.90 ± 0.36 |
|  | Cβ deviations >0.25Å | 0.04% | 0.61% |
|  | Bad bonds: | 0.02% | 0.00% |
|  | Bad angles: | 0.30% | 0.82% |
|  | Cis Prolines: | 0.00% | 0.00% |
| Low-resolution Criteria | CaBLAM outliers | 4.00% | 3.90% |
|  | CA Geometry outliers | 0.91% | 0.96% |
| Additional validations | Tetrahedral geometry outliers | 1 | 1 |

**Supplementary Table 2.** **Novel compounds generated from machine learning.**

| Code | SMILES |
| --- | --- |
| 0 | CC(C)(C)OC(=O)NC(CC(=O)OC(C(=O)C1CC1)C1CC1)c1ccccc1Cl |
| 1 | O=C(O)NCC1CCCCN1C(=O)NC(COc1ccccc1F)C(F)(F)F |
| 2 | COc1ccccc1C(=O)NC(CC(C)C)C(=O)OC(C)C(=O)NC(=O)Nc1ccc(F)cc1 |
| 3 | CCC1(CC)NC(=O)N(CC(C)(O)CN2CCOCC2)C1=O |
| 4 | Nc1nc(CN2CCN(S(=O)(=O)Cc3ccon3)CC2)nc(Nc2ccccc2)n1 |
| 5 | COC(=O)C1CC(C2CCCCC2)CN1S(=O)(=O)c1c(C)cccc1C(=O)NC(C)(C)C |
| 6 | CC1(C)C(O)CCN1C(=O)C(=O)Nc1ccc(OC(F)F)c2ccccc12 |
| 7 | CNC(=O)c1cc(Oc2cc(C)ccc2NS(=O)(=O)c2cnc3n2CCCC3)ccn1 |
| 8 | Cc1ccc2nc(COc3ccccc3C(=O)N3CSCC3C(=O)NCC3CCCC3)cc(=O)n2c1 |
| 9 | COC(=O)C(CNS(=O)(=O)c1ccc(Cl)cc1-c1ccccc1)CC(=O)OC(C)(C)C |
| 10 | CNC(=O)c1cc(S(=O)(=O)N(CC(=O)N2CCCCC2C)C2CCS(=O)(=O)C2)ccc1OC |
| 11 | COC(=O)C(CC(C)(C)C)NC(=O)C(NS(=O)(=O)c1ccc2ccccc2c1)C(C)C |
| 12 | CCC1(CO)CCN(C(=O)C2(NC(C)=O)CCCC2)CC1O |
| 13 | CC(C)C(NC(=O)OC(C)(C)C)C(=O)NC(C(=O)OCc1ccccc1)c1cccnc1 |
| 14 | COC(=O)C(CCF)NS(=O)(=O)c1cccc(C)c1C(=O)NCC(C)C |
| 15 | Cc1c(C(=O)NC(C)C(=O)N2CCCCC2)sc2cccc(F)c12 |
| 16 | COC(=O)CC1(S(=O)(=O)NC(=O)C2(Cc3ccccc3Br)CC2)CC1c1ccc(F)cc1 |
| 17 | COc1ccc(C(=O)N(CCC(=O)OC(C)(C)C)C(=O)OC(C)(C)C)c2ccccc12 |
| 18 | COCCC1(C(=O)NS(=O)(=O)c2ccc3c4c(cccc24)CCC3)CCCCC1 |
| 19 | CCCS(=O)(=O)Nc1cc(NC(=O)C(CC(C)C)NC(=O)OC)c(OC)cc1C |
| 20 | COC(=O)Cc1csc(N(C(=O)C2CC3CCCCC3N2C(=O)c2ccccc2Cl)C(C)C)n1 |
| 21 | COC(=O)CC(NC(=O)C1(C(=O)N(C)C)CCC1)c1cccc(F)c1 |
| 22 | COC(C(=O)N=S(C)(=O)c1c(Cl)cccc1Cl)C(=O)OC(C)(C)C |
| 23 | CCn1c(=O)c2ccc(C(=O)N3CCCCC3c3nnc(CC(F)(F)F)o3)nc2n(C)c1=O |
| 24 | CC(C)(C)NS(=O)(=O)CC(=O)OC(C(=O)Nc1ccccc1-c1ccccc1Cl)C(C)(C)C |
| 25 | COC(=O)C(CC(C)C)S(=O)(=O)CC(=O)NCc1nc2ccccc2n(C(F)(F)F)c1=O |
| 26 | CNC(=O)C1(NC(=O)CC2NC(=O)NC2=O)CCCC1 |
| 27 | COC(=O)NC(C(=O)OCC(=O)N(CCC#N)c1ccccc1)C(C)C |
| 28 | O=C(Cc1ccccc1)N1CCCC1C(=O)N1CCCC1c1nc(Cc2ccccn2)no1 |
| 29 | COC(=O)CC(NC(=O)C1CCCS1(=O)=O)C(=O)Nc1ccccc1C |
| 30 | O=C(CSc1nc(O)c2cnn(-c3ccccc3)c2n1)N(Cc1ccco1)CC1CCCO1 |
| 31 | CCNC(=O)NC1(C(=O)OCC(=O)Nc2ccc(Oc3ccccc3OC)cc2)CCCC1 |
| 32 | COc1ccc(CC(=O)N(CC(=O)N(C)C)CC(F)(F)F)c2ccc(O)c(O)c12 |
| 33 | O=C(C1CCCCN1C(=O)c1c2c(nc3ccccc13)CCC2)N1CCOCC1 |
| 34 | COCCCCN(Cc1ccc(C#N)cc1)C(=O)c1cc(C)[nH]c1C(C)(C)C(=O)O |
| 35 | CCn1ccnc1C(NS(=O)(=O)c1cnc2onc(C(=O)N(C)C)c2c1)C(C)C |
| 36 | CCN(C(=O)c1nc(C(C)C)n(-c2ccc(F)cc2F)n1)C(C)(C)c1nn[nH]n1 |
| 37 | COc1ccccc1-c1ccc(C(=O)NCC2CC(C(N)=O)=NO2)s1 |
| 38 | O=C(OCc1cn(Cc2ccccc2)nn1)C1CCCC(=O)N1Cc1cccnc1 |
| 39 | CC(=O)Nc1ncn(-c2ccc(S(=O)(=O)N3CCCCC3c3nc(=O)n(C)[nH]3)cc2)n1 |
| 40 | CNc1c(Cl)ncnc1NS(=O)(=O)c1ccc(Cl)c(-c2nnnn2CCO)c1 |
| 41 | CC(=O)NC(C)C(=O)OC(C)C(=O)Nc1ccc(Oc2ccccc2Cl)cc1 |
| 42 | NCC1CN(C(=O)Cn2c(-c3cscn3)nc3ccccc32)CC1c1ccccc1F |
| 43 | CC(Oc1ccccc1NC(=O)CC(NC(=O)OC(C)(C)C)C(F)F)C(=O)Nc1nccs1 |
| 44 | Cc1ccc(S(=O)(=O)NC(CC(C)C)C(=O)OCC(CC2CC2)C(=O)O)c2ccccc12 |
| 45 | Cc1ccc(-c2nc(-c3nc(C4COCCN4C(=O)OCc4ccccc4)no3)cs2)n1C |
| 46 | CC(NC(=O)C1C(C)OCC(=O)N1Cc1cccc(F)c1)c1cn[nH]c1 |
| 47 | COC(=O)C1(NS(=O)(=O)c2c(Cl)nc3ccccc3c2F)CC2CCC1C2C(=O)OC(C)C |
| 48 | COC(=O)c1c(NC(=O)c2ncn(C)n2)sc(C(=O)Nc2ccnn2C2CCCC2)c1C |
| 49 | COC(=O)c1cc(C(=O)NCC2(NS(C)(=O)=O)CCCCC2)cc(-c2ccc(OC)cc2)c1 |
| 50 | CNC(=O)C1(C(=O)N2CCC3(CC2)COC(=O)N3)CC1 |
| 51 | CCOc1ccc(-c2nnc(Nc3c(F)cc(C(=O)O)cc3Cl)n2CC(=O)NC)nc1 |
| 52 | COc1ccc(C(=O)N2CCCCC2C(=O)N2CC(O)C2)c2ccccc12 |
| 53 | CCC(C(=O)NC(C(=O)OC(C)(C)C)C(C)C)S(=O)(=O)c1ccc(-c2ccccc2)cc1 |
| 54 | CCC(CC)(C(=O)O)C(=O)N(CC(=O)O)C(C)C(=O)Nc1cccc(F)c1 |
| 55 | CNC(=O)COc1c(C(=O)NC(C(=O)OC)C2CC2)ccc2c(C)c3cc(O)ccc3n12 |
| 56 | CS(=O)(=O)NCC12CCCC1CN(C(=O)C1CCOC1c1cnn(-c3ccccc3F)c1)C2 |
| 57 | COc1ccc2cccc(C(=O)N(CCN(C)C)C3CCS(=O)(=O)C3)c2c1 |
| 58 | COC(=O)C1CC(C)CC(C)N1C(=O)CCC(=O)N1CC(=O)Nc2ccccc21 |
| 59 | CCOC(=O)c1cc(CC)sc1NC(=O)C(CCS(C)(=O)=O)NC(=O)c1ccc(CC)cc1 |
| 60 | COCC(NC(=O)c1cc(S(=O)(=O)Nc2c(F)cccc2F)ccc1Cl)C1CCCO1 |
| 61 | CN(C)C(=O)C(Cc1ccccc1)NC(=O)C(Cc1ccco1)n1nnnc1-c1ccco1 |
| 62 | CCNS(=O)(=O)C1CCN(S(=O)(=O)c2cccc(C)c2NC(=O)C(C)C)C1 |
| 63 | CCC(NC(=O)c1ccc(Cl)cc1NS(=O)(=O)c1ccc(NC(C)=O)cc1)C(=O)OC |
| 64 | COCC(C)(O)CNC(=O)C1CCCCN1C(=O)COc1ccc(CC(C)=O)cc1 |
| 65 | CCOC(=O)c1ccsc1NC(=O)C(=O)N(CC(O)CO)C(C)C |
| 66 | CCOC(=O)C(NC(=O)c1ccc(C(=O)Nc2cccc(C)c2C)cc1O)C(C)C |
| 67 | CCC(CC)(C(=O)O)C(=O)NC1(c2cccc(C#N)c2)CCOC1C(=O)O |
| 68 | CC(C)N(CC(O)COc1ccccc1)C(=O)C(C)(NC(=O)OC(C)(C)C)c1cccnc1 |
| 69 | CCN1C(=O)COC(C(=O)N(CCC(=O)OC)Cc2ccc3c(c2)OCO3)C1c1ccc(C)o1 |
| 70 | CCCN(CC(=O)OC)C(=O)c1cc(CNS(=O)(=O)c2ccc(C)s2)ccc1Cl |
| 71 | CCC(NC(=O)N1CCC(O)(C(=O)O)CC1)C1CCCO1 |
| 72 | CC(C)OC1(CS(=O)(=O)NC(COc2c(Cl)cccc2Cl)C(C)(C)C)CCOC1C |
| 73 | CC(C)=CC(=O)N1CCOCC1C(=O)NC1CC(O)C1 |
| 74 | CCC(CC)NC(=O)c1cc(NC(=O)c2c(C)coc2CC(=O)OC)ccc1C |
| 75 | COC(=O)CC1(CS(=O)(=O)NC(=O)c2cn(-c3ccccc3)nc2CC(C)C)CCCCC1 |
| 76 | CCOC(=O)c1c(-c2ccc(OC)cc2)csc1NC(=O)C(=O)NC(C(=O)O)C(C)C |
| 77 | CCCCS(=O)(=O)Nc1cc(NC(=O)C(CC)S(C)(=O)=O)cc(C(F)(F)F)c1 |
| 78 | Cc1cccc2c(CC(=O)OCC(C)(C)NC(=O)C34CC5CC(CC(O)(C5)C3)C4)c[nH]c12 |
| 79 | COCCn1c(-c2cn[nH]c2)nnc1N(C)C(C)c1nc(C2CC2)no1 |
| 80 | CC(C)N(CCC(=O)O)C(=O)C(Cc1cc(F)ccc1F)NC(=O)c1cccnc1Cl |
| 81 | COC(=O)C(O)C1CCc2ccccc2N1C(=O)C(NC(=O)C1CC1)C(C)O |
| 82 | Cc1ccccc1C(CC(=O)NS(=O)(=O)C1(CO)CCCCC1)c1ccc(F)cc1 |
| 83 | CCOC(=O)CC(NC(=O)C1CCCN1C(N)=O)C(F)(F)F |
| 84 | CCn1c(CNC(=O)C(Cc2cccnc2)NC(=O)c2cccnc2)nc2ccccc21 |
| 85 | O=C(NCC(c1ccccc1)N1CCc2sccc2C1)C1(n2cnnn2)CCOCC1 |
| 86 | COc1ccccc1C(=O)NC(CC(C)C)C(=O)N1CCS(=O)(=O)CC1c1ccncc1 |
| 87 | COc1ccccc1C(=O)NC(CC(C)C)C(=O)OC(C)C(=O)NCC1Cc2ccccc2O1 |
| 88 | CCC(C)C(NC(N)=O)C(=O)N(C)CC(=O)NC(N)=O |
| 89 | CNC(=O)c1cc(N(C)c2ccc(S(=O)(=O)Nc3nc(C)no3)cn2)c(=O)[nH]c1C |
| 90 | CNC(=O)C1CCC(C)N1C(=O)CCC1NC(=O)NC1=O |
| 91 | CCC(C)C(NC(=O)O)C(=O)N(C)C1CC(=O)N(C)C1 |

**Supplementary Data 1.** **Pairwise structure alignment of the homology model (Sec A) and the template (Sec B).**

H = alpha helix,

B = residue in isolated beta-bridge

E = extended strand, participates in beta ladder

G = 3-helix (3/10 helix)

I = 5 helix (pi helix)

T = hydrogen bonded turn

S = bend

: E1 E2 E3

SecA : STT B B SS B TTT SSTTTB GGGEETTEE TTS SS EEE TT

3 :NVTGLFKDCSKVITGLHPTQAPTHLSVDTKFKTEGLCVDIPGIPKDMTYRRLISMMGFKM: 62

*********** **************** *******************************

10 :NVTGLFKDCSKIITGLHPTQAPTHLSVDIKFKTEGLCVDIPGIPKDMTYRRLISMMGFKM: 69

SecB : STT B B S TTTT SSGGGS GGGEETTEE TTS S EEEE

: E1 E2 E3

: H1 E4 E5 E6 E

SecA : TT SB HHHHHHT SSEEEEEEEEEEE SSB S EEEEEEEESSS EEEB E

63 :NYQVNGYPNMFITREEAIRHVRAWIGFDVEGCHATREAVGTNLPLQLGFSTGVNLVAVPT: 122

************************************ ***********************

70 :NYQVNGYPNMFITREEAIRHVRAWIGFDVEGCHATRDAVGTNLPLQLGFSTGVNLVAVPT: 129

SecB : TT SB HHHHHHT SSEEEEEEEEEEE SSB S EEEEEEEESSS EEEB E

: H1 E4 E5 E6 E

:7 E8 E9 H2 E

SecA :EEEE SS EEEEE EE SSGGGGGGGGGSSS B HHHHHHHHHHHHHHHHTTT SS E

123 :GYVDTPNNTDFSRVSAKPPPGDQFKHLIPLMYKGLPWNVVRIKIVQMLSDTLKNLSDRVV: 182

***** *** * ** ************************************** ******

130 :GYVDTENNTEFTRVNAKPPPGDQFKHLIPLMYKGLPWNVVRIKIVQMLSDTLKGLSDRVV: 189

SecB :EEEEESSSEEEEE EE SSSTTGGGSGGGSS B HHHHHHHHHHHHHHHHTTT SS E

:7 E8 E9 H2 E

:10 H3 E11 E12 E13 E14

SecA :EEESSTHHHHHHHTTTB S B SSS SB EEETTTTEEE TTT TT EE S EEE

183 :FVLWAHGFELTSMKYFVKIGPERTCCLCDRRATCFSTASDTYACWHHSIGFDYVYNPFMI: 242

***************************** ******* ******* ** ***********

190 :FVLWAHGFELTSMKYFVKIGPERTCCLCDKRATCFSTSSDTYACWNHSVGFDYVYNPFMI: 249

SecB :EEESSHHHHHHHHTTTB S B SSS SB EEETTTTEEE TTT SS EE S EEE

:10 H3 E11 E12 E13 E14

: H4 H5 H

SecA :ESGGGT SS HHHHHHTS SSS SSHHHHHHHHHHHHHHHHHTS SS SSH

243 :DVQQWGFTGNLQSNHDLYCQVHGNAHVASCDAIMTRCLAVHECFVKRVDWTIEYPIIGDE: 302

**************** ******************************** ********

250 :DVQQWGFTGNLQSNHDQHCQVHGNAHVASCDAIMTRCLAVHECFVKRVDWSVEYPIIGDE: 309

SecB :ESGGGT SS HHHHHHTT SSS SSHHHHHHHHHHHHHHTTTTS TT SS

: H4 H5

:6 E15 E16

SecA :HHHHHHHHHHHHHHHHHHHHHS SEEEEET SS SS S SEEEEEESS STTGGG

303 :LKINAACRKVQHMVVKAALLADKFPVLHDIGNPKAIKCVPQADVEWKFYDAQPCSDKAYK: 362

* * *********** ************************* *****************

310 :LRVNSACRKVQHMVVKSALLADKFPVLHDIGNPKAIKCVPQAEVEWKFYDAQPCSDKAYK: 369

SecB :HHHHHHHHHHHHHHHHHHHHHS SEEEEET SS SSTT SEEEEEESS STT TT

:H6 E15 E16

:E17 H7 E18 E19 E20 E21

SecA :EEE HHHHTTT SSEEEEES SS SSEEEEEE TT SSEEE STT EEEES

363 :IEELFYSYATHSDKFTDGVCLFWNCNVDRYPANSIVCRFDTRVLSNLNLPGCDGGSLYVN: 422

********* * ********************* **************************

370 :IEELFYSYAIHHDKFTDGVCLFWNCNVDRYPANAIVCRFDTRVLSNLNLPGCDGGSLYVN: 429

SecB :EEE HHHHTTT SSEEEEES SS SSEEEEEE TT SSSEEE STT EEEES

:E17 H7 E18 E19 E20 E21

: E22 E23 E24 E25

SecA :SSEEEE SGGGTT EE EEE S SGGG TTS SS SSEEESTTSSS

423 :KHAFHTPAFDKSAFVNLKQLPFFYYSDSPCESHGKQVVSDIDYVPLKSATCITRCNLGGA: 482

************** **************** ******************

430 :KHAFHTPAFDKSAFTNLKQLPFFYYSDSPCE-----------YVPLKSATCITRCNLGGA: 489

SecB :SSEEEE GGGGTT EE EEE S ----------- SSEEESS SSS

: E22 E23 E24 ----------- E25

: H8 E26 H9

SecA : HHHHHHHHHHHHHHHHHHHTTEEEEE TT HHHHHTTT

483 :VCRHHANEYRLYLDAYNMMISAGFSLWVYKQFDTYNLWNTFTR: 525

********** **************** ***************

490 :VCRHHANEYRQYLDAYNMMISAGFSLWIYKQFDTYNLWNTFTR: 532

SecB : HHHHHHHHHHHHHHHHHHHTTEEEEE TT THHHHTTT

: H8 E26 H9


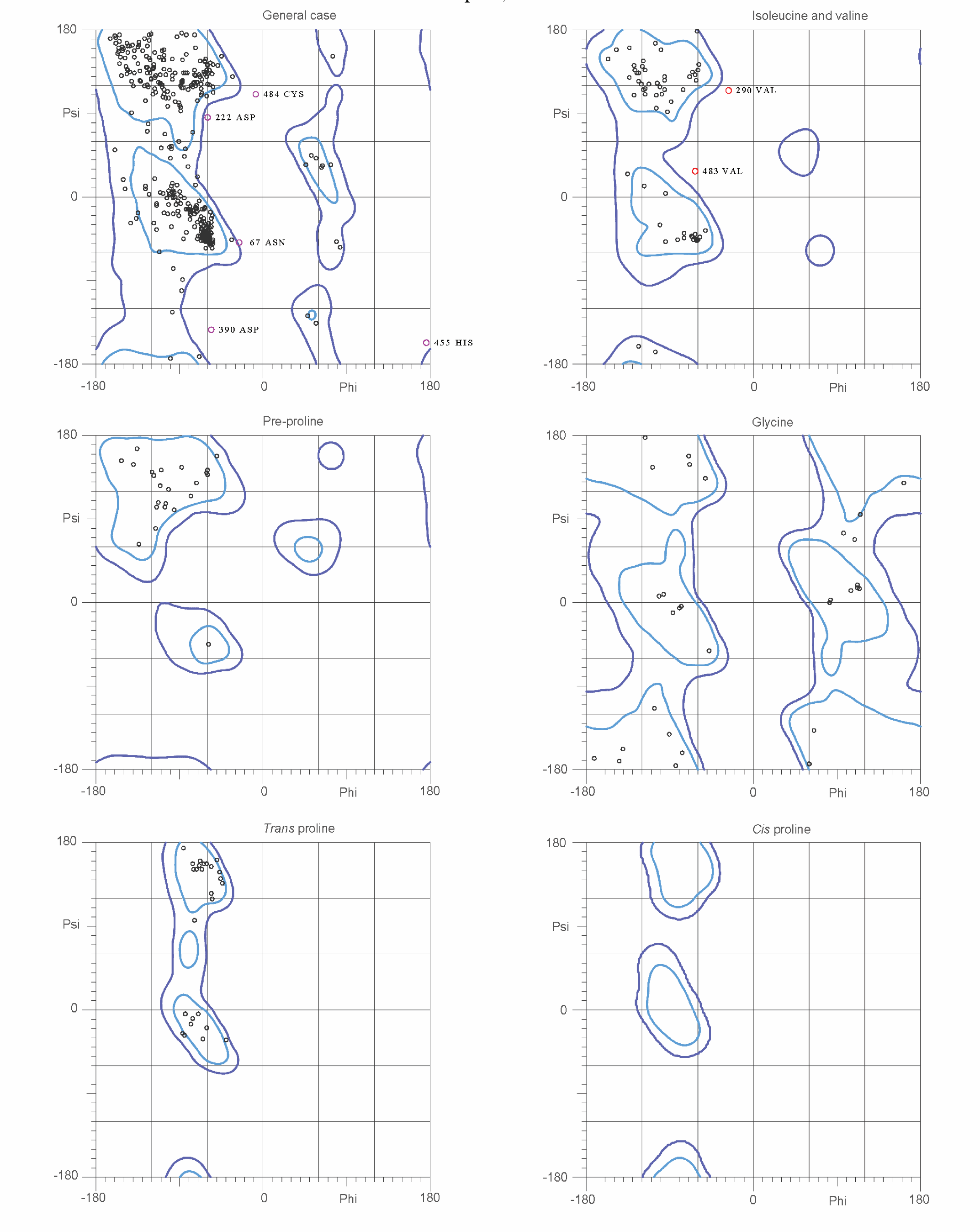


**Supplementary Figure 1. Ramachandran Plots of the homology model.**


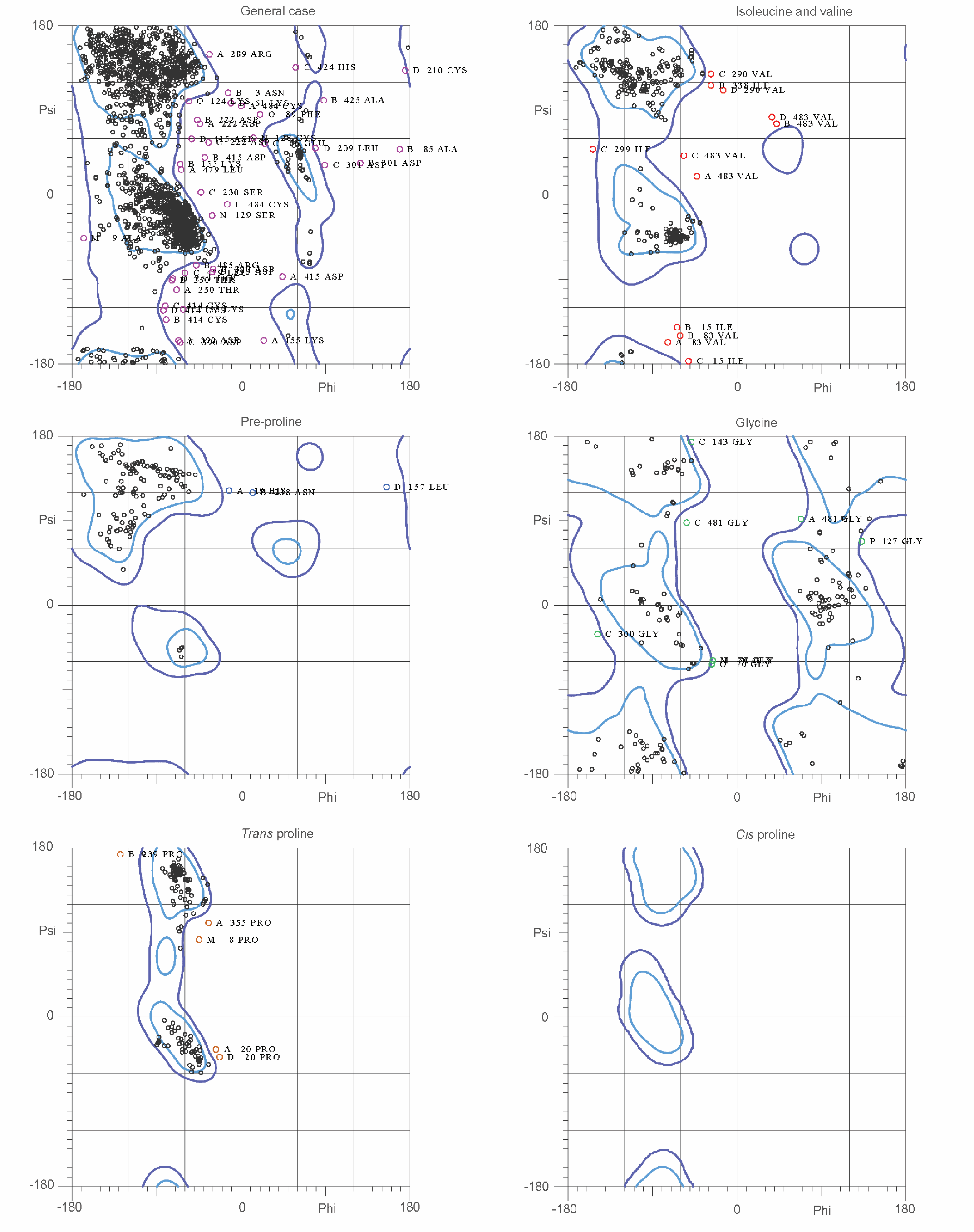


**Supplementary Figure 2. Ramachandran Plots of the template.**


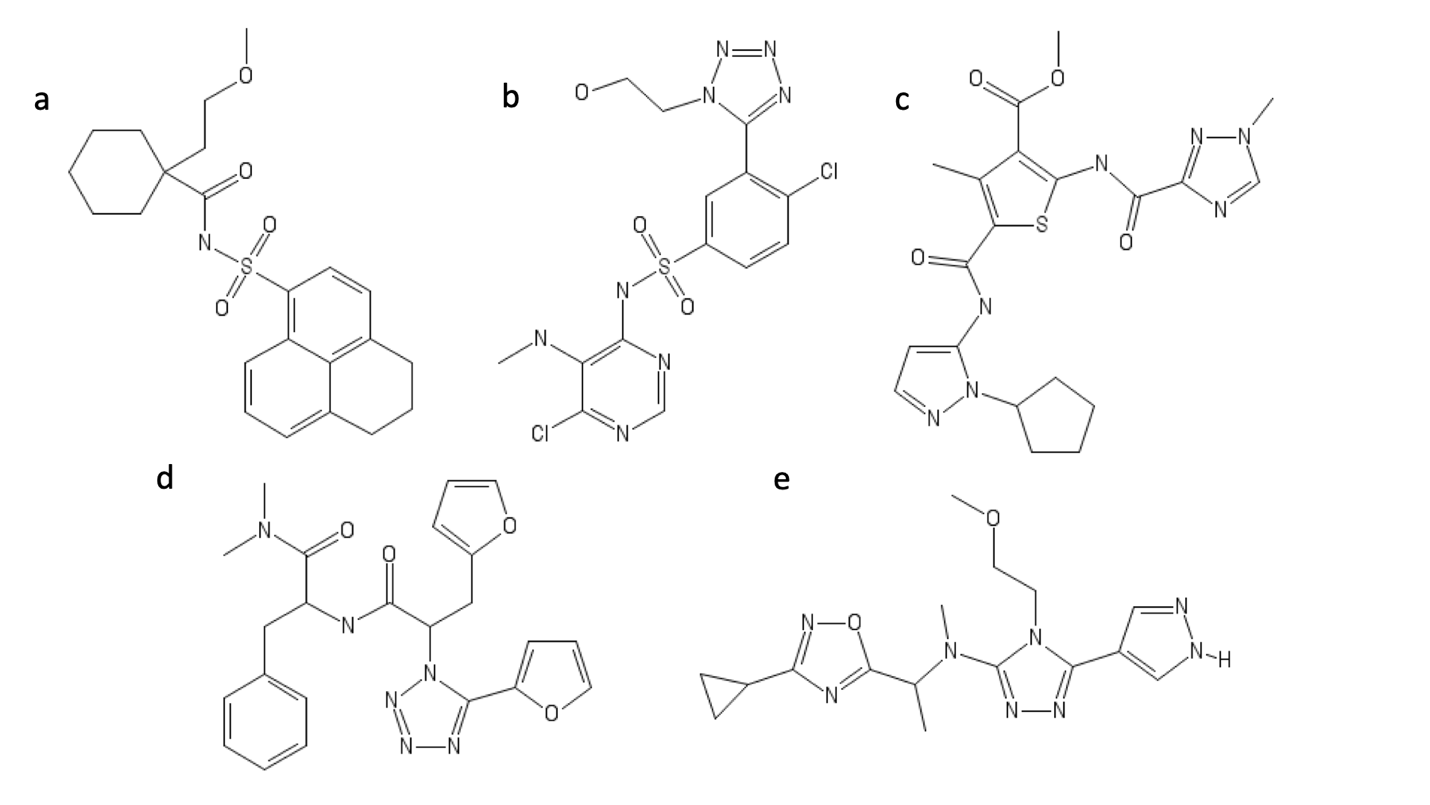


**Supplementary Figure 3**. **Structures of the selected compounds from machine-learning-based modeling with compound code a) 18 b) 40 c) 48 d) 61 e) 79.**
